# Integration of Nutrition Topics in Osteopathic Medical School Curriculum – Shared Experiences

**DOI:** 10.15694/mep.2021.000060.1

**Published:** 2021-03-01

**Authors:** Boris Boyanovsky, Mahboob Qureshi, Samuel Kadavakollu, John Graneto

**Affiliations:** 1 California Health Sciences University College of Osteopathic Medicine

**Keywords:** Integration of Nutrition, Osteopathic medical school curriculum, Preventive care, Systems-based curriculum, Curriculum development

## Abstract

This article was migrated. The article was marked as recommended.

A crucial component of osteopathic medicine’s philosophy is self-regulation, preventive care, and health maintenance, including a healthy lifestyle. More than half of the osteopathic medical graduates pursue a career as primary care physicians, and as such patient education on healthy lifestyle, including eating habits, is a significant part of their daily practice. However, nutrition is often overlooked in the osteopathic medical school curricula. Therefore, strategic inclusion of evidence-based nutrition approaches will equip future osteopathic medical students with an understanding and application of nutrition to the ever-growing primary care medical profession. Working towards this goal, we are implementing nutritional science topics across the pre-clinical years of our new osteopathic medical school’s curriculum. Providing nutrition topics that will fit into the systems-based curriculum may encourage other medical schools to enhance nutrition instruction in osteopathic medical education. Assimilating nutrition as a part of the curriculum will enhance osteopathic medical students’ confidence to counsel and treat their future patient population.

## Introduction

Understanding the human body’s self-healing and health maintenance capability is one of the tenets of osteopathic medicine and a major aspect of osteopathic medical education ( Rogers *et al.*, 2002). Health promotion in all of its aspects, including a healthy lifestyle and nutrition, is crucial for preventing disease. Many osteopathic doctors serve as primary care physicians and coordinate medical care, providing initial assessment, follow-up, and screenings. The primary care physician also provides patient education on various topics, including healthy dietary choices, tobacco cessation, restriction of alcohol consumption, and increase in physical activity. Physicians face several challenges to provide nutrition counseling: visits are short, the providers are infrequently trained in nutrition, and as a result, less than 8% of medical visits touch on this important topic ( Hargrove *et al.*, 2017). Therefore, the need for qualified patient education about lifestyle changes, emphasizing healthy food choices and habits emerges as an integral component of the holistic approach to patient care, a guiding principle of osteopathic medicine.

While nutrition may already be a component of education in some medical schools, it is often overlooked in the osteopathic medical school curriculum. Some practicing osteopathic physicians and residents feel untrained to discuss dietary recommendations, the definitive connection between diet and health with their patients. A study of 257 preclinical students in osteopathic medical schools indicates that most of these students have very little knowledge regarding dietary reference intake, and they are not confident to use nutrition as a potential therapy in their future practice ( Hargrove *et al.*, 2017). Research in allopathic and osteopathic medical schools indicates that more nutrition hours are necessary for the medical school’s curriculum ( Adams, Kohlmeier and Zeisel, 2010), ( Briggs Early, Adams and Kohlmeier, 2015), ( Danek *et al.*, 2017). Since most osteopathic medical students typically enter primary care, the inclusion of nutrition topics in the medical school’s curriculum may help them in their future practice. Besides, the Comprehensive Osteopathic Medical Licensing Examination of the United States (COMLEX-USA) Level 1 dimension II indicates that 12% of the patient presentations for osteopathic medical care come from community health and wellness ( Horber and Gimpel, 2018; Goldgraben *et al.*, 2020).

According to the previous medical colleges’ surveys, only a few provide adequate nutrition education to medical students ( Adams, Butsch and Kohlmeier, 2015), ( Briggs Early, Adams and Kohlmeier, 2015). The nutrition field is quickly evolving, and evidence-based nutrition recommendations should be taught in lieu of the “intuitive” understanding of healthy food choices that are currently expected of our students and future physicians. The addition of a nutrition-related theme across the preclinical year curriculum is a valuable tool to create well-qualified physicians ( Lo, 2000), ( Kushner *et al.*, 1990), ( Adams *et al.*, 2010). Therefore, in our new college, College of Osteopathic Medicine at California Health Sciences University (CHSU), we have undertaken a novel approach of including a practical nutrition component in a teaching kitchen and have integrated nutrition topics throughout our system-based curriculum. Indeed, nutrition-related topics are obvious everywhere in our curriculum. For example, electrolyte balance in physiology; fats, carbohydrates, and protein metabolism in biochemistry, nutrient deficiencies in pathology, fiber and colon cancer, the interrelation between essential fatty acids (α-linolenic acid) and inflammation were introduced during the first two years. In addition, most of the medical biochemistry sessions taught at CHSU have 7-10% of nutrition inclusion throughout the pre-clinical curriculum.

Our goal was to develop and offer a structured, balanced, and comprehensive nutrition theme throughout the osteopathic medical school curriculum. Here we describe our experience and provide a list of nutrition topics ( Table 1) that may correlate with the systems-based curricula of other osteopathic and/or allopathic medical schools. This comprehensive list may help osteopathic medical educators and course directors to integrate nutrition topics in their systems-based curricula to transform nutrition into applied science.

## Evidence-based Nutrition Integrated in a Systems-Based Curriculum

In the following sections, we are briefly describing our conceptualization of how nutrition topics introduced throughout our system-based curriculum may contribute to our students’ holistic education.

## Molecular and Cellular Mechanisms (Biochemistry)

In this course, usually presented early in a system-based curriculum, we emphasize basic principles of nutrition ( Table 1). Here, we introduce the essential micro and macronutrients required to sustain life, water and electrolytes, carbohydrates, fats, proteins, vitamins, and minerals. Along with the metabolism of macromolecules as energy sources for biochemical reactions, we introduce the dietary sources, recommended intakes, and populations at risk for deficiency or excess. Here, we also introduce the role of alcohol as a significant source of “empty calories” and the difference between recommended dietary intakes and the average American intake of foods and nutrients. We also emphasize the importance of energy balance ensuring proper body composition, the functioning of the human body during fed, fasting, and starvation states, the importance of diet and nutrition in maintaining homeostasis, nutrition-related diseases, and health outcomes.

Also, the management of some inborn errors of metabolism, such as phenylketonuria (PKU), can be virtually achieved by diet. However, physicians should be not only aware of foods that patients with PKU must avoid but also be aware that these patients subsist largely on medical food, so social determinants of health and quality of life are issues that come into play when the nutritional management of PKU is discussed with these patients. Metabolism of macromolecules was also introduced in this system. Finally, some of the inborn urea cycle disorders can also be controlled by decreasing the protein amount (or nitrogen consumption) in the diet.

## Musculoskeletal System

In the musculoskeletal system course, we emphasize the role of proteins as building blocks of the human muscular system and energy sources. We emphasize amino acids’ role in major biochemical reactions and pathways, food sources, and recommended intakes of protein foods or protein shakes. Here, we also introduce malnutrition and protein deficiency states (kwashiorkor, marasmus). Students also learn conditions that increase or decrease protein requirements and how protein supplementation needs to change through the life cycle.

An important point to emphasize about cartilage and joint health is the role of diet glycosaminoglycans and to question their benefit as in food additives ( Tannock *et al.*, 2006), ( Reginster *et al.*, 2001). The athletes’ nutrient needs are addressed briefly in the coursework, and the corresponding teaching kitchen component focuses on sports nutrition. The musculoskeletal system course is an ideal place to introduce hydration and dietary supplements both for individuals with more sedentary lifestyles as well as those engaging in intense physical activities.

## Immune System

Although the human immune system has remarkable resilience and is able to ensure proper defense against pathogens even with a highly restricted food supply, it has limitations, and there are nutrition components essential for its proper function. Remarkably, overnutrition may also impair its proper functioning ( Andersen, Murphy and Fernandez, 2016).

Although in modern western societies, starvation is a rare phenomenon, highly restrictive diets, such as vegan diets, may impair the immune system. On the other hand, poor dietary choices leading to obesity and a pro-inflammatory state are also detrimental. Therefore, in this course, we emphasize the importance of balanced macronutrient intake essential for the body’s optimal functioning. In contrast, in developing countries, starvation is a serious challenge, especially in growing individuals. Therefore, the pursuit of comprehensive medical knowledge requires our physicians to be aware of this social malice and to be able to adequately advise and help their patients either recently immigrated to this country or while on a mission abroad.

We believe that understanding the mechanism of true allergic and anaphylactic reactions and knowing the foods that are capable of triggering them can significantly increase the patients’ awareness to prevent many potentially life-threatening episodes. Therefore, food allergies and intolerances, as well as celiac disease, are introduced in the coursework.

## Cardiovascular System

The abundance and the easy access to high-fat, high-calorie food is a hallmark of the Western culture but is also a curse, considering its effects on the cardiovascular system. Dietary fats have long been singled out as a significant cardiovascular disease risk ( Wang and Hu, 2017), ( Siri-Tarino *et al.*, 2010). However, the guidelines for fat intake continue to evolve based on new studies, and physicians must keep current with the recommendations for patient care. Therefore, several important topics related to fat intake and metabolism are also introduced in this course.

First, saturated, and trans-fatty acids have been shown to significantly contribute to cardiovascular disease, while unsaturated fats, particularly omega-3 fatty acids, may play a preventive role for cardiovascular disease development through their anti-inflammatory properties. Therefore, contemporary physicians should know the pharmacotherapies available and the dietary sources of these important nutrients. Regarding lipoproteins, the very low-density lipoproteins (VLDL) and low-density lipoproteins (LDL) are atherogenic, whereas high-density lipoproteins (HDL) transporting cholesterol to the liver for clearance from the body is considered “the good cholesterol” and athero-protective. Although a low-fat diet has a limited effect on LDL and HDL, it may be beneficial in lowering VLDL/triglyceride levels, and patients should be encouraged to consider it, especially the “Mediterranean diet,” which uses mostly vegetable and fish-derived fats rich in omega-3 unsaturated fatty acids ( Benjamin *et al.*, 2019).

Second, weight gain is directly correlated with hypertension. Studies have shown that each pound of weight loss can lower blood pressure by approximately one mmHg. Therefore, fats, a significant source of calories, should be lowered in everyday diet, and foods rich in trans-fats, such as margarine, should be completely excluded. Animal fat (unfortunately bacon) and other foods rich in calories and fats, such as potato and corn chips, should be discouraged.

In conclusion, there is significant evidence for the benefit of dietary interventions on high cholesterol and hypertension. Students at our institution learn about the various commercially available diets and their effectiveness at reducing cardiovascular risk.

## Respiratory System

Although foods have little direct effect on respiratory function, in this course, we emphasize the benefits of a balanced diet, rich in anti-inflammatory foods on overall health. Following a healthy lifestyle may help preventing respiratory diseases, especially in immunocompromised and elderly individuals. Nutrition is extremely important in patients with chronic diseases, including chronic respiratory conditions, such as chronic, recurrent pneumonia, and chronic obstructive pulmonary disease. For example, to help reduce nosocomial infections, glucose and macronutrients should be tightly controlled ( Ingels, Vanhorebeek and Van den Berghe, 2018). The opposite scenario is also important - malnutrition can seriously affect lung functions. Food allergies also may affect respiratory function, and prevention and treatment can be reinforced here. In this system, we also debriefed the common environmental pollutants that cause respiratory dysfunction.

## Renal System

Our curriculum’s renal system presents the opportunity to integrate several important nutrition topics, such as fluid and electrolyte balance, micronutrient homeostasis, caloric balance, protein diet and nitrogen excretion, and the importance of diet in kidney function.

Diet also has a particularly important role in patients suffering from chronic kidney failure. It should be emphasized that most patients with chronic kidney disease need to make dietary changes, and many should be referred to a Registered Dietitian for individualized management and nutrition counseling. These patients must be placed on a diet restricted in potassium, sodium, magnesium, and phosphate ( Jankowska, Rutkowski and Dębska-Ślizień, 2017). Even protein consumption should be monitored carefully. Patients with genetic defects in protein/nucleic acid metabolism should be advised to adhere to a strict diet with balanced protein content. Hyperuricemia is another condition that deserves specific nutrition education for patients.

Fluid balance should be emphasized regarding multiple population groups. Athletes and seasonal workers should be advised on appropriate hydration during heavy exercise or working outside during high temperatures. The role of electrolyte-enriched drinks should be explained in detail, and the myths created by advertisements brought to scientific reality. The role of fluid substitution should also be covered in detail in patients with diabetes insipidus.

The potassium balance should be emphasized in patients with diabetes. Renal tubular acidosis is rare but still deserves nutritional balance, and the introduction of appropriate diets is warranted. Nephrolithiasis is another important condition that can be prevented or improved by a suitable diet. Here, we emphasize the agents alkalizing or acidifying the urine that could trigger or prevent kidney stone formation. For example, a simple increase in fluid intake has proven beneficial in all cases. A study emphasizes that even small increases in fluid intake can reduce new stone formation ( Littlejohns *et al.*, 2020). Prevention of recurrent calcium stones is aimed at decreasing the lithogenic factors (calcium and oxalate) and increasing the concentrations of stone formation inhibitors, such as citrate. Achieving these goals requires dietary modification, including increased fluid intake.

Interestingly, the risk of kidney stone formation might be affected by the type of beverage consumed. For example, coffee, tea, and alcohol have been associated with a lesser risk of stones. Thus, there is no particular evidence that these beverages should be avoided to prevent stone formation. At the same time, cranberry juice is notorious and advertised for decreasing urinary tract infections. Ingestion of moderate amounts is unlikely to be harmful and there is no evidence that this beverage is beneficial for stone prevention. All these studies warrant a detailed education of future physicians to avoid influence by non-scientifically substantiated ideas and improve their ability to apply for evidence-based medicine when educating their patients.

## Gastrointestinal System

The digestive system is another course closely related to nutrition. There is a myriad of examples where diet may be beneficial to patients with various gastrointestinal (GI) tract conditions. Worth mentioning are the vitamin uptake and conditions that may influence it. In this system, we have introduced topics such as digestion and absorption of nutrients and applications of water and lipid-soluble vitamins in medicine.

Gluten enteropathy is a condition that can be successfully controlled through diet. The primary sources we could find dietary gluten are wheat and barley. At a glance, avoiding these components seems easy, but they are included in so many other nutrition additives and pre-made foods that extensive patient education is vital and major lifestyle adjustments are recommended. A few concepts are important to all patients: exclude foods containing wheat and barley; soybean, corn, rice, and potatoes can be consumed if tolerated well. The gluten-free diet includes foods that are naturally gluten-free such as fresh fruits and vegetables, seafood, meat (including poultry), nuts, and dairy products. Information about gluten content is available in the labels of most foods sold at the market, but the patients should be trained on how to find this information and to inquire if in doubt. From alcoholic beverages, beers should be avoided because they could contain gluten. Dairy products (foods produced from milk) may not be well tolerated because many patients may also have lactose intolerance. Oats can be consumed if well tolerated. However, a strict gluten-free diet can result in deficiencies of fibers, vitamins B, and trace minerals ( El Khoury, Balfour-Ducharme and Joye, 2018). In general, patients who are suffering from celiac disease should be referred to a specialized registered dietitian to enhance the patient’s education. All of these important nutritional topics related to the GI system are included in this system.

Finally, current dietary trends, such as the Paleo Diet and the Ketogenic Diet (very high protein, low carbohydrate diets), may impact the gut microbiome’s health. While overall, this is a rapidly evolving area of research, popular diets should be introduced to medical students to be conversational about these with patients in the future.

## Endocrine and Reproductive Systems

Many metabolic diseases could be prevented and maintained through healthy nutritional choices and diets. A “stand-alone” topic where nutrition plays an important role in prevention and management is type II diabetes. In fact, lifestyle change was found to be more effective than metformin at reducing the progression of prediabetes to diabetes ( Ackermann *et al.*, 2008). Lifestyle interventions can improve healthy weight management, decrease hemoglobin A1c levels, maintain blood glucose levels, and improve pregnancy and birth outcomes. Nutrition interventions are in the management guidelines for many metabolic conditions, put forth by the American Diabetes Association, and physicians should be proficient in lifestyle interventions for patients with insulin resistance, metabolic syndrome, and other dysglycemias.

Obesity is an epidemy comparable to tobacco and alcohol abuse in regard to poor health outcomes. Obesity has been associated with decreased life expectancy, atherosclerosis, diabetes, osteoarthrosis, non-alcohol fatty liver, and some cancers ( Blüher, 2019). Importantly, strict diets may influence the reproductive health of women. This topic was incorporated into this course.

## Neurological System and Neurology

Nutrition may be beneficial in neurological conditions, such as dementia, and a ketogenic diet may be used in the management of epilepsy reviewed by Gupta *et al*. ( Gupta *et al.*, 2017). Students and physicians should be able to discuss nutrition adjustments with patients or individuals at high risk. Eating disorders, heavy metal toxicities, and the effects of hyper-palatable foods on the reward system are all areas that are covered during neurological system courses. The instruction and hands-on teaching classes that go along with these may allow students to appreciate diets for patients with neurological conditions and present these options to their future patients. Finally, the critical relation between mood, behavior, and food is also included in this course.

## Mechanism of Disease (Pathology)

Nutrition plays a crucial role in various diseases’ pathophysiology. Hence, we have included topics such as nutritional deficiency, absorption disorders and prevention, malnutrition, weight management, and health promotion and disease prevention; nutrition in cancer ( Table 1).

The role of nutrition in genetic conditions, such as enzymatic deficiencies (inborn errors of metabolism), is complex, but the application of a system-level approach may help the students understand the principles of maintaining the balance in these patients.

Indeed, a healthy diet and preventive care could markedly protect the human body against chemical, biological, and physical stressors that we are exposed to on a daily basis. Basic research skills required to conduct nutritional or health promotion related research is also included in this course.

**Table 1.** Example of a systems-based curriculum with integrated nutrition topics

Course	Suggested topics
**Molecular and Cellular Mechanisms (Biochemistry)**	•Principles of Nutrition • Water and Electrolytes • Metabolic Fuels and Dietary Components • Macronutrients: Carbohydrates, Proteins, and Fats • Nutrients Essential to Healthy Tissues • Alcohol and Dietary Fibers • Energy Metabolism in Fed and Fasting State • Energy Balance and Body Composition • Nonessential Trace Minerals • Nutrition and Human Metabolism
**Musculoskeletal System**	• Proteins: Deficiency, Food Sources, and Requirements • Diet and Weight Management • Nutrition Related to Sports • Hyperuricemia and Gout
**Immune System**	• Nutrition and Immunity (Caloric and Protein Deficiency) • Food Allergies and Reactions • Veganism and Vegetarianism
**Cardiovascular System**	• Fatty Acids and Lipoproteins • Foods Contributing to Cardiovascular Diseases • Hypertension and Other Risk Factors Cardiovascular Diseases • Physical Activity • Weight Loss Strategies
**Respiratory System**	• Nutrition and Respiratory Health (Malnutrition in Respiratory Disease and Pulmonary Disorders)
**Renal System**	• Micronutrients: Major Minerals and Fluids • Caloric Balance-Exercise • Kidney Stones • Renal Failure and Diet
**Gastrointestinal System**	• Digestion, Absorption, and Transport of Nutrients • Dietary Supplements • Fat-Soluble Vitamins • Water-Soluble Vitamins • Nutrition in Liver Diseases • Gastrointestinal Tract Illnesses • Gut-Brain Interactions and Feeding Behavior • Diarrhea, Water, Electrolyte, Acid-Base Balance • Nutritional Anemias • Disorders Associated with Nitrogen Excretion
**Endocrine and Reproductive System**	• Hormonal Regulation of Nutrient Metabolism • Diabetes: Nutritional Mechanisms and Dietary Management • Life Cycle Nutrition: Pregnancy, Fetal, and Infant Nutrition • Life Cycle Nutrition: Toddlers Through Adolescents and Older Adults • Growth and Development • Obesity • Bone Health (Osteoporosis)
**Neurological System**	• Nutritional Neuroscience (Ammonia Toxicity) • Heavy Metal Poisons • Eating Disorders (Nutritional Psychiatry) • Nutrition and Eye • Antioxidants (Types, Sources, Effects) • Cognitive Decline
**Mechanism of Disease (Pathology)**	• Nutritional Deficiency Diseases • Absorption Disorders and Prevention • Amino Acids as Nutritional Supplements • Cancer Nutrition: Molecular Mechanisms, Prevention and Treatment, and Primary Prevention • Malnutrition • Nutrition of Surgery, Trauma, Infection, and Wound Healing • Food-Bone Disease • Nutrition Associated with Inborn Errors of Metabolism • Drug-Nutrient and Supplement-Nutrient Interaction • Weight Management • Health Promotion and Disease Prevention

## Discussion and Conclusions

In this commentary, we have delineated a systematic way of integrating nutrition science topics into the systems-based osteopathic medical school curriculum. We have reaffirmed the significance of this integration as suggested by other studies ( Adams *et al.*, 2010; Adams, Kohlmeier and Zeisel, 2010; Kushner *et al.*, 2014; Martin *et al.*, 2020). Medical schools are working towards emphasizing nutrition in their curricula. However, few colleges of medicine have sustained nutrition programs, and even fewer provide the number of hours slated to be included in the nutrition curriculum. Our goal is not to create registered dietitians or physicians specialized in nutritional sciences. However, physicians are held in high regard by patients and their recommendations on adequate lifestyle changes have a high impact on improving health outcomes ( Hargrove *et al.*, 2017). Physicians especially osteopathic physicians should initiate the discussion on healthy lifestyle habits with patients and then consider referring them to registered dietitians, counselors, and life coaches, as needed, so the patient can make sustainable changes. Evidence suggests that much of these efforts is a team effort of healthcare specialists. Therefore, we are including healthcare students from other disciplines (nursing, dietetics, pharmacy) to facilitate interprofessional education.

We believe that we offer a novel and unique approach through the integration of nutrition in our coursework and application in the teaching kitchen. As such, we will be routinely collecting data on medical students’ knowledge, attitude, and beliefs related to nutrition in their personal lives, how they intend to care for their patients in the future, and to what extent they foresee including nutrition inpatient care.

## Take Home Messages

• There is a potential advantage of integrating nutrition topics throughout a system-based curriculum instead of offering an independent and/or elective course to create physicians with more comprehensive knowledge about evidence-based nutritional approaches to patients counseling and treatment.
• Including nutrition science in osteopathic medical curricula will contribute to the patient’s holistic approach by improving the future osteopathic physicians’ knowledge and skills to apply nutrition as preventive measure and treatment.
• The comprehensive collection of topics in the systems-based courses may help medical educators across the globe who are interested in nutrition instruction time in their curricula.
• Nutritional sciences learning is more effective through multi-professional engagement (biomedical science, clinical science, and dietetics specialists).

## Notes On Contributors

**John Graneto**, DO, M.Ed., FACOP, FACOEP-dist, FNAOME, Dean, Chief Academic Officer, Vice President of Health Affairs, and Professor at California Health Sciences University College of Osteopathic Medicine, Clovis, CA.

**Mahboob Qureshi**, MD, MPH, Ph.D., Associate Dean of Academic Affairs and Professor at California Health Sciences University College of Osteopathic Medicine, Clovis, CA.

**Boris Boyanovsky**, MD, Ph.D., is an Associate Professor of Biomedical Education at The California Health Sciences University College of Osteopathic Medicine, Clovis, CA.

**Samuel Kadavakollu**, Ph.D., M.Sc. Associate Professor of Biomedical Education and Director of MCAT and Preparatory Programs at California Health Sciences University College of Osteopathic Medicine, Clovis, CA.

*BB and MQ contributed equally to this work.

## Declarations

The author has declared that there are no conflicts of interest.

## Ethics Statement

Ethics Approval was not required since this article is a personal view or opinion piece.

## External Funding

This article has not had any External Funding
