# Supplementary material for: Integration of Nutrition Topics in Osteopathic Medical School Curriculum – Shared Experiences
Source: MedEdPublish (2016). 2021 Mar 1;10:60. [Version 1] doi: 10.15694/mep.2021.000060.1 (PMC10939619; doi:10.15694/mep.2021.000060.1)
Supplement: Peer review history

# Reviewer report: Reviewer response for version 1

**DOI:** 10.21956/mep.18955.r27131

**Published:** 2021-10-06

**Authors:** Julie Hunt

**Affiliations:** 1 Lincoln Memorial University

This review has been migrated. The reviewer awarded 4 stars out of 5 In this article, the authors offer their opinion that additional nutritional education should be present in the curriculum of osteopathic doctors, and potentially allopathic doctors. The introduction does a good job of framing up the reasons for their opinion. The authors list, by organ system, what nutritional topics are incorporated into that unit. While they do not provide references for most of their statements about nutrition, many of the facts will be familiar to readers who are trained physicians or medical educators. The inclusion of a table listing these topics was helpful.This article may be of interest to those who design or evaluate medical school curricula, as it provides a potential way to incorporate nutrition into systems-based courses.

Reviewer Expertise:

NA

No decision status is available

# Reviewer report: Reviewer response for version 1

**DOI:** 10.21956/mep.18955.r27130

**Published:** 2021-07-30

**Authors:** P Ravi Shankar

**Affiliations:** 1 American International Medical University

This review has been migrated. The reviewer awarded 4 stars out of 5 I enjoyed reading this article. The authors provide practical suggestions which seem justified, in my opinion, regarding introducing a comprehensive nutrition curriculum during an organ system-based undergraduate osteopathic medicine course. Their suggestions seem equally relevant for undergraduate medical courses. Nutrition, often, does not get the attention it deserves. We had offered an online nutrition course to our undergraduate medical students in Aruba and Saint Lucia to supplement their knowledge. The authors put forward their arguments in a logical manner. Their outline of a nutrition course arranged according to organ systems may be useful for other educators in planning and delivering similar courses.

Reviewer Expertise:

NA

No decision status is available

# Reviewer report: Reviewer response for version 1

**DOI:** 10.21956/mep.18955.r27129

**Published:** 2021-03-02

**Authors:** Deb Halder

This review has been migrated. The reviewer awarded 3 stars out of 5 This paper is a short communique on the issues of curriculum designing regarding the intended fields of the authors. The introductory part has covered the proper literature reviews on the issues of the topic. The introductory part has also dealt with the aims and objectives of the paper.The gravest concern of this paper is that it could not provide with all justified literature to prove their ground. The insufficiency of dealing with literature to justify their imposition has made the arguments loose and disconnected.

Reviewer Expertise:

NA

No decision status is available
